# Co-Reactant Engineering for Au Nanocluster Electrochemiluminescence

**DOI:** 10.3390/molecules30244748

**Published:** 2025-12-12

**Authors:** Nguyen Phuc An Khang, Joohoon Kim

**Affiliations:** 1 Department of Chemistry, Research Institute for Basic Science, Kyung Hee University, Seoul 02447, Republic of Korea; 2 KHU-KIST Department of Converging Science and Technology, Kyung Hee University, Seoul 02447, Republic of Korea; 3 Department of Information Display, Kyung Hee University, Seoul 02447, Republic of Korea

**Keywords:** co-reactants, gold nanoclusters, electrochemiluminescence, co-reactant engineering strategy

## Abstract

Co-reactants are essential in co-reactant-based electrochemiluminescence (ECL) systems because they generate reactive intermediates that can oxidize or reduce ECL luminophores, thereby driving ECL emission. In the context of ECL, gold nanoclusters (Au NCs) have emerged as innovative luminophores, owing to their tunable electronic structures and excellent biocompatibility. However, their efficiency in ECL applications is often compromised by challenges such as limited excited-state generation and non-radiative losses. To tackle these practical challenges, advanced co-reactant engineering strategies have been developed to improve the performance of Au NCs in ECL systems. This review begins with a brief overview of the mechanisms of ECL. Subsequently, a systematic overview of various co-reactant engineering strategies is presented, including: (1) using innovative co-reactants to replace traditional ones due to their lower toxicity and better biocompatibility; (2) applying co-reaction accelerators to reduce the onset potential and improve the production of reactive intermediates from co-reactants; (3) combining co-reactants with luminophores or creating integrated nanostructure assemblies of co-reactants, co-reaction accelerators, and luminophores to achieve shorter electron transfer paths and reduced energy loss for stable high-intensity ECL emission; (4) utilizing host-guest strategies that encapsulate co-reactants within cavities to stabilize radical intermediates and minimize environmental quenching. This review provides a comprehensive overview of recent developments in co-reactant engineering for Au NCs-based ECL systems, thereby encouraging further exploration and understanding of these systems and expanding their potential applications.

## 1. Introduction

**Figure 1:**
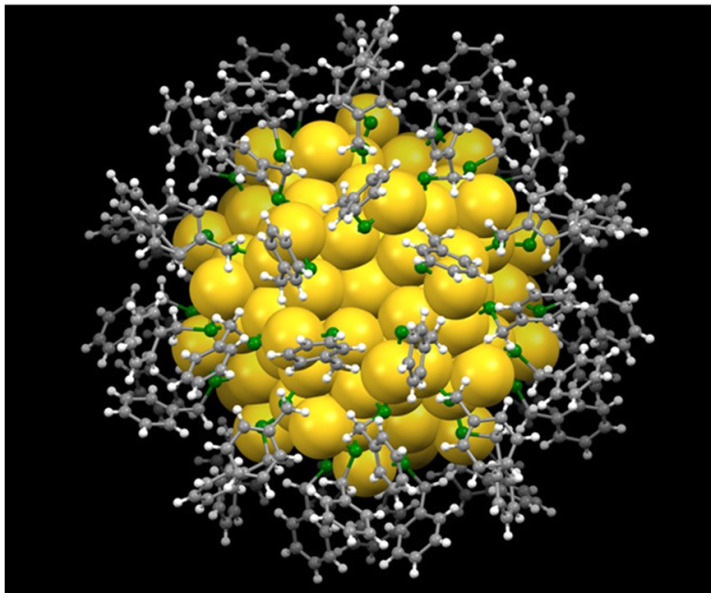
Atomic structure of the Au_144_(SCH_2_Ph)_60_ NC resolved by X-ray crystallography. Yellow, Au; green, S; gray, C; white, H. Copyright 2018 The American Association for the Advancement of Science [17].

In recent decades, gold nanoclusters (Au NCs) have attracted increasing attention because of their unique luminescence properties, which differ from those of larger counterparts due to their ultra-small sizes comparable to the Fermi wavelength of an electron [1,2,3,4]. Au NCs have thus been used in a wide range of applications across fields such as sensing, catalysis, environmental science, and medicine [3,4,5,6,7,8,9,10,11]. Specifically, Au NCs are nanoscale particles, usually protected by thiolate ligands on their surface, with a core diameter of less than 2 nanometers, placing them between small molecules and metal nanoparticles (NPs) [6,12]. The core–shell structured Au NCs consist of a few to several hundred gold atoms in a metallic core, surrounded by a shell of protective ligands that prevent aggregation and maintain stability, as shown in Figure 1. Unlike larger gold nanoparticles (Au NPs), which exhibit surface plasmon resonance, Au NCs behave more like molecules, showing distinct HOMO-LUMO electronic transitions, high photoluminescence, and tunable fluorescence that spans from visible to near-infrared [12,13,14,15]. They also offer additional benefits such as high photostability and excellent biocompatibility [16].

Electrochemiluminescence, also known as electrogenerated chemiluminescence (ECL), is a light-emitting phenomenon that occurs electrochemically at electrode surfaces [18,19]. It involves generating excited states of luminophores via electrochemical processes. In the electrochemical processes, highly reactive intermediates (free radicals) are generated and undergo electron-transfer reactions to form excited states of ECL luminophores, which then emit light as they relax and return to the ground state [20,21,22,23,24]. The phenomenon was first observed in 1908 by C. G. Schluederberg during the electrolysis of sulfuric acid [25,26]. Over the past century, ECL has gradually evolved into an effective and powerful analytical technique, owing to its advantages such as high sensitivity, high spatiotemporal controllability, simple instrumentation, and low background noise [27,28,29,30,31]. Specifically, because of these advantages, ECL has been widely used in sensing and imaging analyses, with a strong ability to detect various target analytes, such as protein biomarkers [32,33], metal ions [34,35], small molecules [36,37], nucleic acids [38,39], and cells [40,41].

Among various ECL luminophores, Au NCs have gained recognition as innovative luminophores due to their high versatility in tuning luminescent properties, along with their distinctive physicochemical features such as high photostability and biocompatibility [42,43,44,45,46,47]. Recent advances have demonstrated that Au NCs-based ECL systems can be integrated into practical sensing, medical testing. At the same time, their facile and mild synthetic procedures, often using inexpensive thiolate ligands and ambient conditions, demonstrated that Au NCs could be a cost-effective alternative to more complex or expensive organometallic ECL emitters. However, their practical use in ECL systems is often compromised by challenges such as limited excited-state generation and non-radiative losses [48,49,50]. To address the practical issues, substantial efforts have been made to develop innovative co-reactants that serve as essential components in co-reactant-based ECL processes. Co-reactants are chemical species that participate in ECL reactions by generating reactive intermediates through electrochemical oxidation or reduction, which can react with luminophores to produce the excited state [18]. Depending on whether they are oxidized or reduced at the electrode, they can be classified as either anodic or cathodic co-reactants [18]. Through their involvement in these electron transfer processes, co-reactants critically influence the ECL emission properties. Recent studies show that strategic selection and modification of co-reactants can significantly improve ECL intensity, stability, and spectral properties [51,52,53], thereby overcoming long-standing challenges such as low Φ_ECL_ and limited tunability of Au NCs-based ECL systems. For example, the structural modification of the classical co-reactant triethylamine (TEA) was demonstrated by replacing the ethyl groups in TEA with isopropyl groups to create (diisopropylamino)ethanol (DIPEA), and further adding a hydroxyl group to DIPEA to produce DIPEA-OH [51]. Benefiting from the isopropyl substitution and hydroxyl addition to TEA, co-reactant-mediated low-potential ECL of Au NCs was achieved, which enhanced the ECL intensity and Φ_ECL_ of the Au NCs at a low potential of 0.75 V. In this regard, a comprehensive review of recent advances in this field is both timely and necessary.

This review aims to highlight advanced co-reactant solutions, emphasizing both traditional and innovative co-reactant strategies, utilizing novel cathodic and anodic co-reactants, co-reactant catalysis, and synergistic interactions between co-reactants and nanoclusters. First, we provide foundational knowledge on ECL and Au NCs-based ECL systems, followed by a systematic overview of co-reactants used with Au NCs. Next, we present a comprehensive overview of recent co-reactant engineering strategies that improve the ECL performance of Au NCs, including the design of new co-reactant molecules, co-reactant accelerators, structural integration, and host-guest strategies. Finally, we offer an objective perspective on current challenges and future opportunities for further co-reactant engineering research.

## 2. Fundamentals of Au NCs-Based ECL

### 2.1. ECL Mechanisms of Au NCs

**Figure 2:**
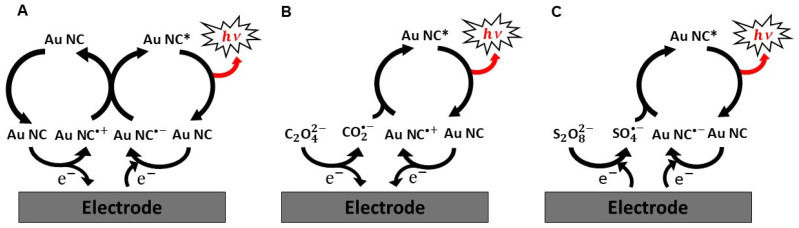
Different pathways in the ECL mechanism: (**A**) annihilation pathway, (**B**) oxidative–reduction pathway, and (**C**) reductive–oxidation pathway.

The mechanistic pathways of Au NCs-based ECL are generally understood to follow the typical ECL mechanism of traditional luminophores. The ECL process of Au NCs can be classified into two sub-pathways that promote ECL, often referred to as the annihilation and co-reactant pathways [54]. In the annihilation pathway, both oxidized and reduced Au NC species are generated electrochemically, either by alternating electrode potentials for redox reactions at the same electrode or by applying redox potentials at two closely spaced electrodes [18]. For example, as illustrated in Figure 2A, both the oxidized ${Au\;NC}^{•+}$ and the reduced ${Au\;NC}^{•-}$ can be electrochemically generated by applying alternating pulsed potentials at the same electrode surface (Equations (1) and (2), respectively), and then these oxidized and reduced species diffuse and recombine according to an ion annihilation reaction in the vicinity of the electrode surface. This homogeneous electron transfer process between ${Au\;NC}^{•+}$ and ${Au\;NC}^{•-}$ generates the electronically excited state Au NC* (Equation (3)), which then relaxes to the ground state and emits light (Equation (4)).

(1) $$Au\;NC-e^{-}\to {Au\;NC}^{•+}\;\left(\mathrm{oxidation}\;\mathrm{at}\;\mathrm{electrode}\right)$$

(2) $$Au\;NC+e^{-}\to {Au\;NC}^{•-}\;\left(\mathrm{reduction}\;\mathrm{at}\;\mathrm{electrode}\right)$$

(3) $${Au\;NC}^{•+}+{Au\;NC}^{•-}\to {Au\;NC}^{*}+Au\;NC\;\left(\mathrm{excited}\text{-}\mathrm{state}\;\mathrm{formation}\right)$$

(4) $${Au\;NC}^{*}\to Au\;NC+hv\;\left(\mathrm{light}\;\mathrm{emission}\right)$$

In contrast, the co-reactant pathway involves the electrochemical formation of only the oxidized ${Au\;NC}^{•+}$ or the reduced ${Au\;NC}^{•-}$ by applying a single potential step or sweeping the potential in one direction in the presence of sacrificial reagents called co-reactants. The co-reactants are chemical species that produce highly reactive intermediates capable of reacting with the oxidized ${Au\;NC}^{•+}$ or the reduced ${Au\;NC}^{•-}$ during electrochemical oxidation or reduction, leading to the formation of the excited state Au NC*. The corresponding co-reactant pathways are referred to as “oxidative–reduction” and “reductive–oxidation” ECL, respectively [25,55]. The oxidative–reduction and reductive–oxidation routes are also often called anodic and cathodic ECL, respectively, to emphasize the electrochemical nature of the excitation process and the polarity of the working electrode involved in the luminophore’s activation [18]. Typical co-reactants include oxalate, peroxydisulfate, tripropylamine (TPrA), 2-(dibutylamino)ethanol (DBAE), and benzoyl peroxide (BPO). As a typical example of the oxidative–reduction pathway, as shown in Figure 2B, the oxalate ion C_2_O_4_^2−^, which can produce a strong reductant upon oxidation, and Au NC are both oxidized at the electrode surface by applying an anodic potential step or sweep, generating ${CO}_{2}^{•-}$ radical anion (a strong reductant) and ${Au\;NC}^{•+}$ (Equations (5) and (6), respectively). Then, ${CO}_{2}^{•-}$ reduces ${Au\;NC}^{•+}$ to form the excited state ${Au\;NC}^{*}$ (Equation (7)), which relaxes back to the ground state accompanied by light emission (Equation (8)).

(5) $$C_{2}O_{4}^{2-}-e^{-}\to {[C}_{2}O_{4}^{•-}]\to {CO}_{2}^{•-}+{CO}_{2}\;\left(\mathrm{oxidation}\;\mathrm{at}\;\mathrm{electrode}\right)$$

(6) $$Au\;NC-e^{-}\to {Au\;NC}^{•+}\;\left(\mathrm{oxidation}\;\mathrm{at}\;\mathrm{electrode}\right)$$

(7) $$Au\;{NC}^{•+}+{CO}_{2}^{•-}\to {Au\;NC}^{*}+{CO}_{2}\;\left(\mathrm{excited}\text{-}\mathrm{state}\;\mathrm{formation}\right)$$

(8) $$Au\;{NC}^{*}\to Au\;NC+hv\;\left(\mathrm{light}\;\mathrm{emission}\right)$$

On the contrary, the reductive-oxidation pathway is conducted by applying a cathodic potential step or sweep to reduce both the co-reactant and the Au NC luminophore. For example, as shown in Figure 2C, the peroxydisulfate ion $S_{2}O_{8}^{2-}$ and the Au NC are both reduced at the electrode surface, generating ${SO}_{4}^{•-}$ radical (a strong oxidant) and ${Au\;NC}^{•-}$(Equations (9) and (10), respectively). Then, ${SO}_{4}^{•-}$ oxidizes ${Au\;NC}^{•-}$ to form the excited state Au NC* (Equation (11)), followed by ECL emission through relaxation (Equation (12)).

(9) $$S_{2}O_{8}^{2-}+e^{-}\to {[S}_{2}O_{8}^{•-}]\to {SO}_{4}^{•-}+{SO}_{4}^{2-}\;\left(\mathrm{reduction}\;\mathrm{at}\;\mathrm{electrode}\right)$$

(10) $$Au\;NC+e^{-}\to {Au\;NC}^{•-}\;\left(\mathrm{reduction}\;\mathrm{at}\;\mathrm{electrode}\right)$$

(11) $$Au\;{NC}^{•-}+{SO}_{4}^{•-}\to {Au\;NC}^{*}+{SO}_{4}^{2-}\;\left(\mathrm{excited}\text{-}\mathrm{state}\;\mathrm{formation}\right)$$

(12) $$Au\;{NC}^{*}\to Au\;NC+hv\;\left(\mathrm{light}\;\mathrm{emission}\right)$$

Although the annihilation ECL pathway appears simple and straightforward, not requiring additional co-reactants, both oxidized and reduced species must be stable enough to coexist in the same proximity to the electrode. This requirement often restricts the annihilation pathway to using organic solvents because the potential window of water is not wide enough to generate both stable ${Au\;NC}^{•+}$ and ${Au\;NC}^{•-}$ species on most electrode materials [56]. Additionally, the annihilation pathway requires using pulsed electrode potentials or two closely spaced electrodes to generate both the ${Au\;NC}^{•+}$ and ${Au\;NC}^{•-}$, which limits its practical use in Au NCs-based ECL. In contrast, as an alternative, the co-reactant pathway offers notable advantages because it can operate in aqueous environments and utilizes unidirectional potential steps and sweeps. These benefits result in stronger ECL intensities, lower background noise, and improved compatibility with miniaturized and portable devices. Therefore, the co-reactant pathway is especially beneficial for Au NCs-based ECL.

Along with the mechanistic pathways of Au NCs-based ECL described above, it is also crucial to understand how ECL is used in practical applications, at least in its simplest and most accessible forms. The most straightforward application of ECL is in analytical sensing, where light from an electrochemically excited luminophore is used to quantify target analytes. This approach is most commonly used in commercial clinical diagnosis, especially in automated immunoassay platforms [22]. The most fundamental and widely used ECL application requires two instrumental components, including electrochemical and optical parts [57]. For more detail, the setup consists of a standard electrochemical workstation equipped with a three-electrode system (working, counter, and reference electrodes), combined with a photon-detection module such as a photomultiplier tube (PMT) or a charge-coupled device (CCD) camera. When an appropriate potential is applied, the electrode generates excited-state luminophores that emit light, which is captured by the detector and correlated with analyte concentration. This simple instrumental configuration demonstrates why ECL has become one of the most practical and robust signaling strategies for chemical and biological sensing applications.

### 2.2. Co-Reactants in Au NCs-Based ECL Systems

The first observation of ECL emission from Au NCs was reported in 2011 by Zhu et al. using K_2_S_2_O_8_ as the co-reactant, marking a significant advancement in the ECL field [58]. They described a reductive-oxidation ECL pathway of Au NCs in the presence of K_2_S_2_O_8_ to generate the excited state Au ${NCs}^{*}$ for dopamine sensing applications. They also demonstrated that the co-reactant pathway dominates the annihilation by revealing the relatively instability of ${Au}_{25}^{•-}$ and ${Au}_{25}^{•+}$ intermediates in the ECL process [58]. Since then, many other interesting studies on the ECL of Au NCs have been conducted, focusing on co-reactant pathways with popular co-reactants such as TPrA [59,60,61], triethanolamine (TEOA) [62,63], and potassium persulfate (K_2_S_2_O_8_) [64,65]. While these initial works mainly concentrated on developing Au NC luminophores suitable for efficient Au NCs-based ECL systems using various reducing or stabilizing agents such as lipoic acid (LA), bovine serum albumin (BSA), methionine (Met), glutathione (GSH), N-acetyl-L-cysteine (NAC), and others, many innovative advances in co-reactant engineering have recently gained significant attention. This is due to the crucial roles that co-reactants play in ECL pathways, which boost ECL intensity and diversify ECL wavelengths [42,59]. For example, Zhuo et al. demonstrated the color-tunable ECL from individual Au NCs confined in a porous hydrogel matrix by adjusting the concentration of triethylamine (TEA) co-reactant [52]. They observed that when the TEA concentration changes, the surface-related ECL of Au NCs exhibits a continuous, color-tunable ECL ranging from 625 to 829 nm, while the core-related ECL remains steady at 489 nm. This difference arises because the surface ECL originates from ligand-associated Au(I) surface states that are highly responsive to redox processes triggered by co-reactants, whereas the core emission reflects the cluster’s stable internal structure. Mechanistic studies revealed that the color-tunable ECL of the hydrogel-confined Au NCs results from the dynamic surface reconstruction of Au NCs caused by reactive TEA radicals. But the detailed understanding of how TEA interacts with Au NCs to modulate surface-related ECL remains to be explored. Besides the role of co-reactants in reconstructing Au NCs surfaces to tune emission color, Yuan et al. further emphasized that the co-reactant’s stability and its interaction with the environment are crucial in dictating the overall ECL efficiency and spectral output [66]. These findings emphasize the critical roles of co-reactants in ECL systems, particularly in systems involving Au NCs. Table 1 provides a comprehensive overview of recent advances in the use of co-reactants for Au NCs-based ECL systems. The table highlights some key ECL features, including ECL pathways and ECL quantum yields (Φ_ECL_), for various co-reactants in Au NCs-based ECL systems. The Φ_ECL_ represents the efficiency of light emission generated through electrochemical reactions, defined as the ratio of photons emitted to the number of redox events, in the ECL process [18]. This is different from the fluorescence quantum yield (Φ_FL_), which is defined as the ratio of the photons emitted to the number of photons absorbed in a purely photophysical process [67]. Despite substantial progress with co-reactants, challenges remain, such as achieving high Φ_ECL_, which requires developing advanced co-reactant engineering techniques to further improve and optimize their performance.

## 3. Co-Reactants Engineering Strategies

Recently, many notable strategies have been developed to address the existing challenges in Au NCs-based ECL systems, such as low Φ_ECL_, lack of universal co-reactants, and limited compatibility and stability for practical applications. Therefore, this section provides an overview of the recently reported strategies, which include four main directions. The first is the design of new co-reactant molecules. The second focuses on the use of co-reactant accelerators. The third involves the integration of co-reactants with nanoclusters. And finally, host-guest encapsulation strategies.

### 3.1. Design of New Co-Reactant Molecules

**Figure 3:**
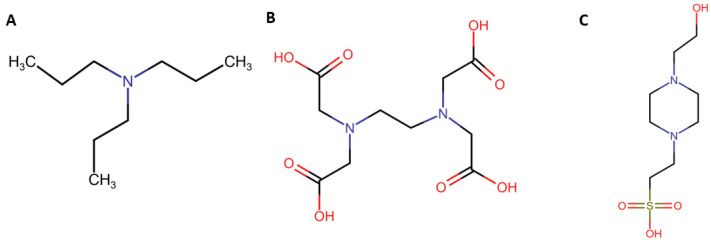
Chemical structures of (**A**) TPrA, (**B**) EDTA, and (**C**) HEPES.

Throughout the development of fundamental research and practical applications of ECL, various co-reactants have been introduced to enhance performance and expand options in ECL systems [25,85,86]. One of the most classic and widely used co-reactants is TPrA (Figure 3A), which serves as an anodic co-reactant for the traditional ECL system based on Ru(bpy)_3_^2+^. TPrA, which has a tertiary amine structure, can form a strong reductive radical after the deprotonation at the alpha carbon, followed by its electrochemical oxidation [87]. Its effectiveness in the oxidative-reduction ECL pathway has also made it a popular choice in Au NCs-based ECL systems. Despite its popularity, TPrA exhibits high toxicity, strong pH dependence, and moderate water solubility, which limit its use in physiological environments [88]. Along with TPrA, various amine molecules have been investigated as anodic co-reactants, with their effectiveness steadily increasing from primary to secondary amines, and tertiary amines being the most efficient [89]. The pKa values of amines and the pH of the amine solution are also critical factors influencing the oxidative-reduction ECL pathway [90]. Based on these findings, recent studies have broadened the range of co-reactants for Au NCs-based ECL systems by discovering co-reactant molecules with superior properties. For example, ethylenediaminetetraacetic acid (EDTA), whose structure is shown in Figure 3B, has been widely used as a chelating agent due to its excellent chelating properties. Additionally, because it contains two tertiary amine groups and four carboxyl groups, with the pKa values of the amine groups being 6.13 and 10.37, respectively, EDTA can serve as a co-reactant similar to TPrA but with lower toxicity, better solubility, and pH tunability. Wang et al. reported the use of chelating agent EDTA as a novel co-reactant for enhancing near-infrared ECL from lipoic acid (LA)-stabilized Au NCs (LA-Au NCs) at physiological pH levels [83]. In their study, metal ions, particularly Mg^2+^, effectively impact the ECL signal through complexation with EDTA and interaction with the LA-Au NCs. Moreover, the chelation process contributed to increased structural stability of the LA-Au NCs-EDTA system, promoted the formation of effective reductive sites, and suppressed side reactions, further improving the performance of the ECL system.

Furthermore, building upon the previous study, Wang et al. proposed the application of a commonly used biological buffer 4-(2-hydroxyethyl)-1-piperazineethanesulfonic acid (HEPES) (Figure 3C) for the LA-Au NCs system, with the aim of further enhancing near-infrared ECL performance [84]. They found that the presence of different types of metal ions can modulate the ECL emission of LA-Au NCs with HEPES as an anodic co-reactant. Specifically, Zn^2+^ was shown to enhance the ECL of the LA-Au NCs-HEPES system, whereas Mg^2+^ and Ca^2+^ reduced the ECL signal. The enhancement of Zn^2+^ was mediated by its specific coordination interactions with the Au NCs and HEPES, forming Au NCs-Zn^2+^-HEPES intermediates. This coordination facilitated more efficient formation of the excited state, leading to a dramatic increase in ECL intensity. In contrast, Ca^2+^ and Mg^2+^ exhibited weaker coordination with Au NCs and HEPES, resulting in no significant ECL enhancement under similar conditions. These illustrate the differences in their binding affinities, showing that Zn^2+^ has the strongest interaction, followed by Ca^2+^ and Mg^2+^. The novelty of this study lies in the dual role of HEPES, as it functions both as a buffer and as a co-reactant. HEPES not only maintains pH stability but also enhances ECL through its interaction with metal ions, leveraging the lone electron pair on the nitrogen atom, similarly to ammonia or other amines. Furthermore, HEPES presents a biocompatible alternative to TPrA, which is recognized for its toxicity and instability. Interestingly, when comparing HEPES and EDTA as anodic co-reactants in the LA-Au NCs system, the ECL response was optimal at physiological pH, indicating that HEPES is more suitable than EDTA for relevant applications.

Besides using biological buffers or chelating agents, structural modifications—such as replacing ethyl groups with more electron-donating isopropyl groups or hydroxyl functionalities in classical co-reactant TEA—can also lower the oxidation potential and improve the formation and reactivity of radical intermediates, as discussed in the introduction. For example, Chen et al. modified TEA by replacing the ethyl groups with isopropyl groups, producing *N*,*N*-diisopropylethylamine (DIPEA), with the aim of developing a low-potential ECL system [51]. It is well known that hydroxyl groups can facilitate the oxidation of amines, thereby enhancing ECL efficiency [91]. Building on this concept, the Chen group also introduced a hydroxyl group into DIPEA, denoted as 2-(diisopropylamino)ethanol (DIPEA-OH). The use of these co-reactants was demonstrated with BSA-Au NCs. Accordingly, the BSA-Au NC/DIPEA-OH ECL system achieved improved energy efficiency at a lower potential of 0.75 V. Compared to the BSA-Au NC/TEA, the ECL intensity and Φ_ECL_ with DIPEA-OH as a co-reactant increased 22.34-fold and 13-fold, respectively [51]. The use of DIPEA-OH was also demonstrated with the herceptin-encapsulated Au NCs (HER-Au NCs) for the high-performance electrochemiluminescence immunoassay (ECLIA) [92].

### 3.2. Use of Co-Reaction Accelerator

Considering the co-reactant pathway of ECL, co-reactants undergo electrochemical oxidation or reduction to produce highly reactive intermediates. These intermediates, in turn, react with oxidized or reduced forms of Au NCs, leading to the generation of the excited state of the NCs. Consequently, the intensity of the ECL emission is proportional to the number of intermediates formed during this process [93]. In basic terms, increasing the concentration of co-reactant can enhance the production of intermediates and thus elevate ECL emission [94]. However, due to the co-reactant’s limited solubility and the non-linear relationship between its concentration and the number of intermediates, simply raising the concentration will not necessarily lead to a proportional increase in ECL intensity [95]. Therefore, instead of simply controlling the co-reactant’s concentration, researchers have explored co-reaction accelerators to promote the conditions that favor the formation of reactive intermediates and thereby enhance ECL output, leading to more sensitive and effective analytical applications of Au NCs-based ECL systems. Co-reaction accelerators are species that preferentially react with a co-reactant to effectively transform it into strong oxidative or reductive intermediates, while also promoting the production of more intermediates [95,96,97,98,99,100,101]. The co-reaction accelerators are often composed of small molecules or unique nanomaterials [102].

**Figure 4:**
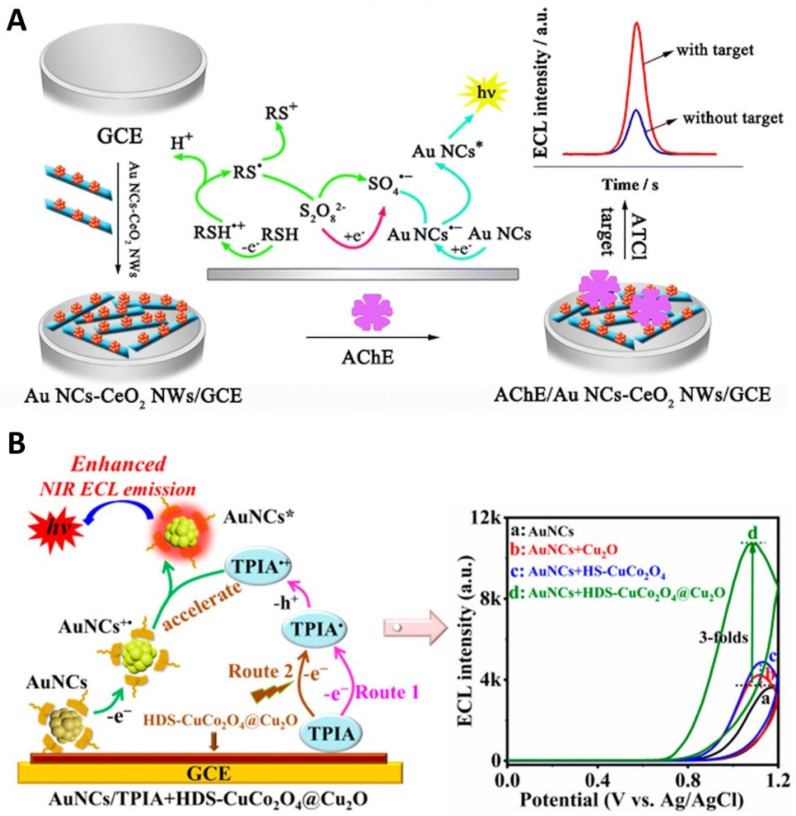
(**A**) Schematic illustration of the fabrication process of an AChE-based ECL biosensor using the Au NCs-S_2_O_8_^2−^ system for ECL generation. Copyright 2018 Springer Nature [103]. (**B**) Schematic illustration of using hollow double-shell CuCo_2_O_4_@Cu_2_O heterostructures as an efficient co-reaction accelerator to enhance the near-infrared ECL of the L-Met-templated Au NCs-TPIA system. Copyright 2022 American Chemical Society [104].

Zhang et al. reported a highly sensitive ECL biosensor for detecting acetylthiocholine (ATCI) using an acetylcholinesterase (AChE) [103]. ATCI was catalyzed by AChE to produce thiocholine, serving as a co-reaction accelerator to enhance the ECL of Au NCs-S_2_O_8_^2−^ system, thereby achieving a low detection limit of 0.17 nM for ATCI. The AChE-based ECL biosensor was constructed using the composites of CeO_2_ nanowires (CeO_2_ NWs) and Au NCs, as shown in Figure 4A.

Jia et al. also reported the highly branched Cu_2_O electroplated on ITO as a co-reaction accelerator for detecting procalcitonin (PCT), using BSA-templated Au NCs as a low-potential cathodic ECL luminophore and K_2_S_2_O_8_ as a co-reactant [105]. The Cu_2_O co-reaction accelerator catalyzed the reductive conversion of K_2_S_2_O_8_ to produce more ${SO}_{4}^{•-}$ anionic radical intermediates. The ${SO}_{4}^{•-}$ can oxidize ${Au\;NC}^{•-}$ for generating Au NCs*, thereby doubling the ECL intensity to meet the requirements of trace analysis. This use of Cu_2_O as a co-reaction accelerator thus enabled the ultrasensitive detection of PCT with a low detection limit of 2.90 fg/mL. Similarly, Wei et al. reported using a Cu_2_S snowflake as a co-reaction accelerator for immunosensing of PCT, employing the same BSA-templated Au NCs as a low-potential anodic ECL luminophore but using TEA as a co-reactant instead of K_2_S_2_O_8_ [106]. The Cu_2_S co-reaction accelerator facilitated the production of more TEA**^•^**^+^ cationic radical intermediates, which enhanced the ECL intensity for trace analysis of PCT with a low detection limit of 2.36 fg/mL. Wei et al. also utilized Fe_2_O_3_ nanoarrays (Fe_2_O_3_ NAs) as a well-ordered co-reaction accelerator for ultrasensitive detection of the tumor biomarker CYFRA21-1, using polypeptide-biomineralized Au NCs as an anodic ECL luminophore and tris(3-aminoethyl)amine (TAEA) as a co-reactant [80]. The use of Fe_2_O_3_ NAs enhanced the electron transfer and energy transmission, forming a higher-energy-state TAEA/Au NCs* from TAEA**^•^**/Au NCs^+^ state intramolecularly. This intramolecular co-reaction acceleration resulted in a linear ECL response across a broad range from 10 fg/mL to 100 ng/mL and a low detection limit of 1.33 fg/mL.

More recently, unique heterostructures have been demonstrated to be effective co-reaction accelerators, amplifying the ECL of Au NCs. Wei et al. proposed the use of hollow double-shell CuCo_2_O_4_@Cu_2_O heterostructures as an efficient co-reaction accelerator to enhance the near-infrared ECL of L-methionine (L-Met)-templated Au NCs with tri-isopropanolamine (TPIA) as a co-reactant (Figure 4B) [104]. The hollow double-shell CuCo_2_O_4_@Cu_2_O heterostructures facilitated the formation of sufficient TPIA**^•^**^+^ cationic radical intermediates to react with the Au NCs cationic radicals, enhancing the near-infrared ECL response of the Au NCs and thus enabling the ultrasensitive immunoassay of the biomarker CYFRA21-1 with a very low detection limit of 0.67 fg/mL. In addition, Wang et al. demonstrated L-histidine-modified zeolitic imidazolate framework-8 (L-His-ZIF-8) as a co-reaction accelerator for encapsulating Au NCs [107]. The L-His-ZIF-8 co-reaction accelerator enhanced electron transfer through its catalytic effect on the electrochemical reduction of $S_{2}O_{8}^{2-}$ co-reactant to generate ${SO}_{4}^{•-}$ anionic radical intermediates sufficiently. Additionally, the spatial confinement effect of L-His-ZIF-8 enabled the encapsulation of Au NCs within its internal cavity, thereby suppressing non-radiative transition losses of excited Au NCs*. The synergistic effects of L-His-ZIF-8 thus enhanced the ECL of Au NCs by improving both electron transfer and radiative transitions, achieving quantitative detection of pro-gastrin-releasing peptide (Pro-GRP), a tumor marker for the diagnosis of small cell lung cancer. These studies highlighted the potential of such heterostructures as effective co-reaction accelerators for Au NCs-based ECL systems.

### 3.3. Integration of Co-Reactant with Au NCs

In conventional co-reactant ECL pathways, the short lifetime of co-reactant radical intermediates can lead to issues with ECL generation during mass transfer between electrochemically generated intermediates due to a long electron transfer path in intermolecular ECL reactions [79]. The efficient ECL generation from Au NCs-based systems with co-reactants can also face challenges due to the complexities of mass transfer between Au NCs and co-reactant intermediates during the limited lifetime of the radical intermediates involved in ECL production. This often requires a high excess of co-reactants for practical applications of Au NCs-based ECL systems [59,108,109]. Intracluster ECL reactions between Au NCs and co-reactants can tackle the issues by reducing the long electron transfer distances needed for intermolecular ECL reactions [110,111,112]. This approach emphasizes the significance of addressing mass transfer challenges between reaction intermediates for achieving efficient ECL performance in Au NCs-based ECL systems.

**Figure 5:**
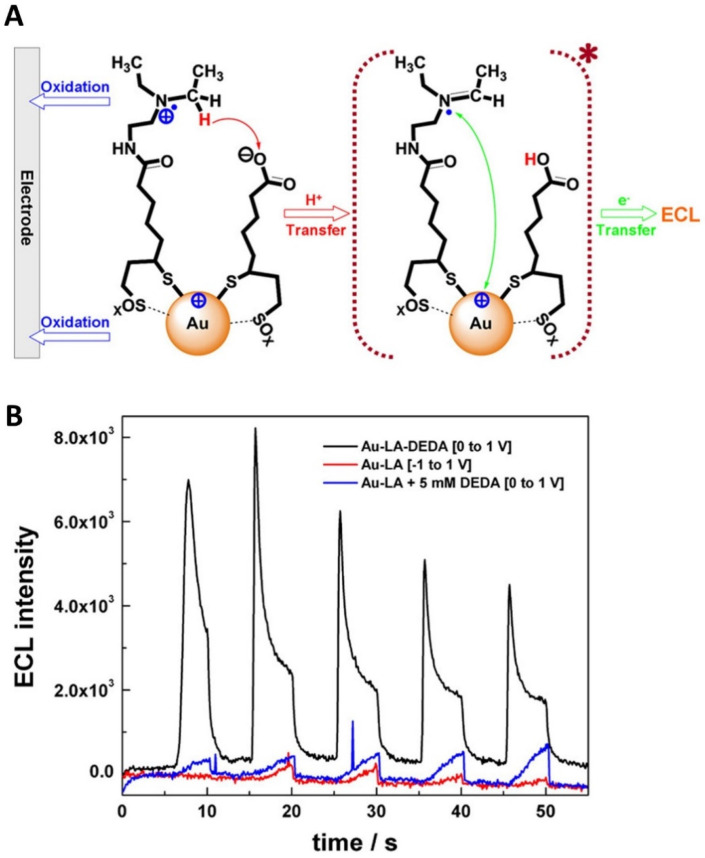
(**A**) Schematic illustration of stepwise ECL generation of Au-LA-DEDA NCs ECL system. The star symbol (“∗”) indicates an excited state. (**B**) Comparison of ECL behaviors of Au-LA NCs with different DEDA configurations: with DEDA covalently attached (black), without co-reactant (red), and with DEDA (blue) in solution. Copyright 2021 American Chemical Society [78].

To achieve the intracluster ECL reactions, co-reactants can be integrated with Au NCs to form binary ECL systems. Wang et al. proposed a binary Au NC-based ECL system by covalently attaching *N*,*N*-diethylethylenediamine (DEDA) co-reactants onto lipoic acid-stabilized Au NCs (Au-LA NCs) (Figure 5A) [78]. They claimed that the binary design reduced the complication of mass transfer between reaction intermediates during their lifetime in the conventional ECL generation pathway. Figure 5B illustrates representative improved ECL traces generated through the intracluster ECL reactions with the binary Au-LA-DEDA system compared to conventional intermolecular ECL systems. The ECL of the binary Au-LA-DEDA system was about 17 times higher than that of the conventional Ru(bpy)_3_^2+^/TPrA ECL system. Wei et al. also reported an efficient ECL label by integrating *N*,*N*-diisopropylethylenediamine (DPEA) co-reactants onto thioctic acid-capped Au NCs (Au-TA NCs) via covalent linkage as a self-enhanced Au NCs (Au-DPEA NCs) [81]. The self-enhanced Au-DPEA NCs were highly effective in improving the ECL of the Au-TA NCs as a binary intracluster ECL system by shortening the electron transfer distance. By combining a multi-site landing DNA walker for signal amplification, the self-enhanced Au-DPEA NCs were used to construct an ECL aptasensor for highly sensitive detection of mucin 1 (MUC1) targets in the range from 1 fg/mL to 1 ng/mL, with a detection limit as low as 0.54 fg/mL. This work demonstrated a synergistic approach that combines the binary Au-DPEA NCs ECL label with an additional signal amplification method, thereby broadening the use of binary ECL cluster systems to develop highly sensitive ECL sensing platforms for clinical applications.

**Figure 6:**
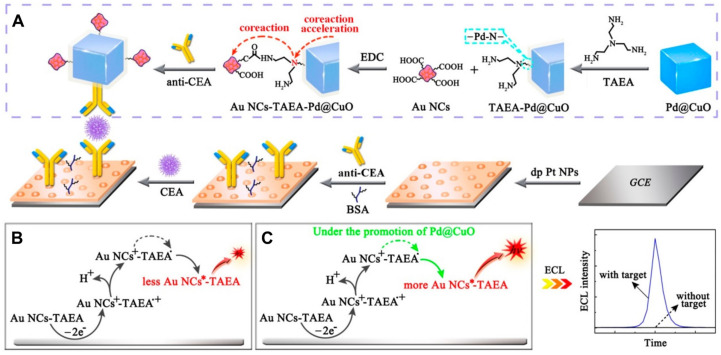
(**A**) Schematic illustration of the ECL immunosensor based on the ternary Au NCs-TAEA-Pd@CuO ECL system. Possible ECL mechanisms of (**B**) only Au NCs-TAEA and (**C**) ternary ECL nanostructure with Pd@CuO as the co-reaction accelerator for the detection of target CEA. Copyright 2018 American Chemical Society [79].

In addition to the binary ECL cluster systems, ternary nanostructures of Au NCs were also demonstrated as highly efficient ECL labels. For instance, Yuan et al. developed a ternary nanostructure of Au NCs that serves as an ECL label for ultrasensitive ECL immunoassays of carcinoembryonic antigen (CEA) [79]. This ternary ECL cluster system was designed by integrating BSA-templated Au NCs, which function as the ECL luminophore, with TAEA and Pd@CuO nanomaterials serving as the co-reactant and the co-reaction accelerator, respectively. This led to the formation of a covalently linked ternary Au NCs-TAEA-Pd@CuO system (Figure 6A). The ternary nanostructure of Au NCs was proposed to function as follows: upon oxidation, both Au NCs and TAEA in the ternary nanostructure were oxidized to form Au NCs^+^-TAEA**^•^**^+^, followed by the loss of a proton from the tertiary amine of TAEA**^•^**^+^ to generate Au NCs^+^-TAEA**^•^** intermediate. Importantly, Pd@CuO in the ternary nanostructure acted as a co-reaction accelerator, enhancing intramolecular electron transfer and energy transmission between Au NCs and TAEA in the Au NCs^+^-TAEA**^•^** intermediate. This led to more efficient formation of the excited state Au NCs*-TAEA, which emitted highly intense ECL compared to the binary Au NCs-TAEA counterparts (Figure 6B,C). The use of the ternary Au NCs-TAEA-Pd@CuO system thus achieved a highly sensitive ECL immunoassay for measuring carcinoembryonic antigen (CEA) antigen with a detection limit as low as 16 fg/mL.

**Figure 7:**
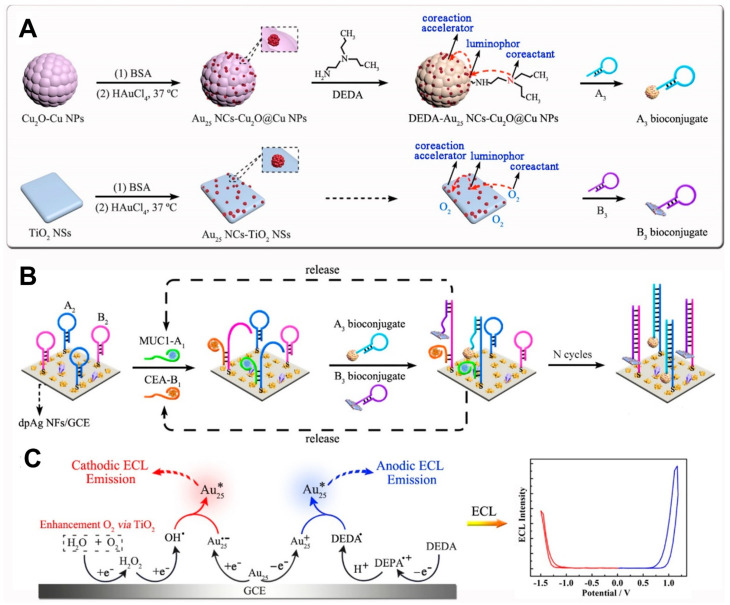
Schematic illustration of the ECL aptasensor based on the BSA-stabilized Au_25_ NCs (Au_25_ NCs) as a dual-polar ECL probe: (**A**) synthesis process of A_3_ bioconjugate with anodic ECL probe (DEDA-Au_25_ NCs-Cu_2_O@CuNPs) and B_3_ bioconjugate with cathodic ECL probe (Au_25_ NCs-TiO_2_ NSs). (**B**) Working principle of the ECL aptasensor for simultaneous detection of MUC1 and CEA. (**C**) Possible ECL mechanisms of simultaneous anodic and cathodic ECL emissions of Au_25_ NCs. Copyright 2019 American Chemical Society [113].

The binary/ternary ECL cluster systems have also been proposed for simultaneous cathodic and anodic ECL emissions of BSA-stabilized Au_25_ NCs (Au_25_ NCs) as a dual-polar ECL probe [113]. The dual-polar ECL emission of Au_25_ NCs was demonstrated for the simultaneous detection of two biomarkers, CEA and MUC1, by employing different co-reactants and co-reaction accelerators to construct binary/ternary ECL cluster systems tailored to the target biomarkers. As shown in Figure 7, the Au_25_ NCs ECL probe was incorporated with Cu_2_O@Cu nanoparticles (Cu_2_O@Cu NPs) and DEDA as anodic co-reaction accelerators and co-reactants, respectively, to construct a ternary nanostructure. The Au_25_ NCs ECL probe was also integrated with TiO_2_ nanosheets (TiO_2_ NSs) as cathodic co-reaction accelerators to form a binary nanostructure. During anodic potential scanning, Cu_2_O@Cu NPs catalyzed the electrochemical oxidation of DEDA co-reactants to enhance the anodic ECL of Au_25_ NCs in the ternary nanostructure. Conversely, during cathodic potential scanning, TiO_2_ NSs promoted the electrochemical reduction in dissolved O_2_ co-reactants to stimulate the cathodic ECL of Au_25_ NCs in the binary nanostructure. Therefore, with continuous anodic and cathodic scanning, the simultaneous detection of the dual biomarkers CEA and MUC1 was achieved, with detection limits of 0.43 pg/mL and 5.8 fg/mL, respectively. This work is significant as a method for achieving dual-biomarker ECL detection without the cross-reactions of dual-luminophores that are possible in traditional dual-biomarker detection.

**Figure 8:**
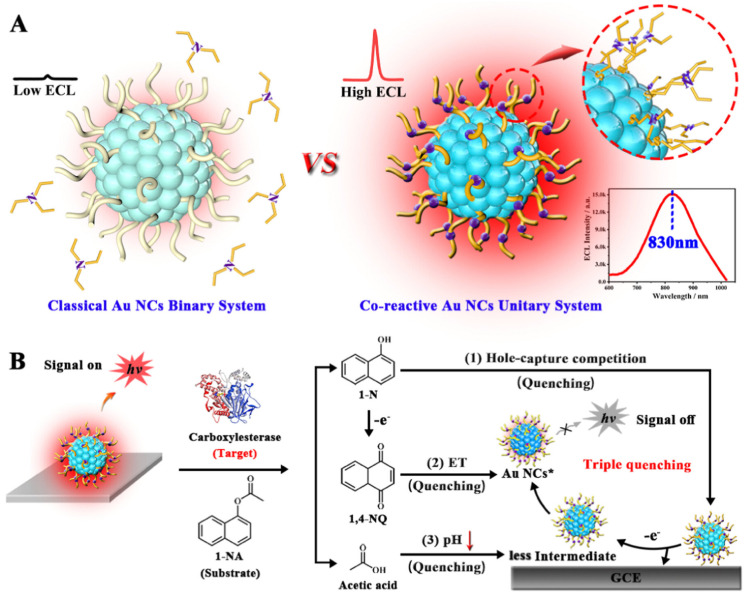
(**A**) Schematic illustration comparing classical ligand-protected Au NCs in a binary system (left) and co-reactive ligand-engineered Au NCs in a simple unitary system (right). (**B**) Schematic illustration of the signal-off ECL platform for CE detection using the unitary DEAET–Au NCs ECL system. Copyright 2024 American Chemical Society [53].

Although the Au NCs-based binary and ternary ECL systems offer significant advantages, they often entail complex preparation and operational procedures. This complexity may ultimately compromise both simplicity and reproducibility in their application. Recently, Zhou et al. reported a simplified co-reactant engineering strategy employing 2-(diethylamino)ethanethiol (DEAET) as a co-reactive ligand to construct a co-reactive unitary Au NCs system for ultrasensitive monitoring of carboxylesterase (CE) activity [53]. As shown in Figure 8, the co-reactive ligand DEAET not only acts as a stabilizer, like traditional ligands, but also functions as a co-reactant to ensure a confinement effect, thereby shortening the charge transfer distance and increasing the local concentration. The resulting DEAET-Au NCs thus exhibited stable anodic ECL performance that surpassed those of traditional Au NCs/TEA and even Ru(bpy)_3_^2+^/TPrA systems without requiring exogenous co-reactants, demonstrating them as a unique Au NCs-based unitary ECL system. For practical application, the DEAET-Au NCs were utilized as a novel near-infrared ECL emitter in a label-free ECL sensor for detecting CE activity, achieving impressive results, including a low detection limit of $9.1\times 10^{-7}U/L$. Therefore, this co-reactive ligand engineering strategy provided a promising approach for ultrasensitive and convenient ECL platforms.

It is also important to recognize that the binary and ternary Au NCs-based ECL systems have unique advantages and limitations that should be taken into account for practical applications. Binary systems offer improved ECL intensity and sensitivity by reducing electron transfer distances and trapping radical intermediates, though their preparation can be relatively complex. Ternary systems achieve the highest ECL performance and offer advanced functionalities, such as dual-polar or multiplexed detection, but generally require more complex synthesis and operational steps. The recently developed unitary co-reactive ligand system combines high ECL efficiency with operational simplicity, making it a promising approach for ultrasensitive and user-friendly ECL sensing platforms. This comparison highlights the trade-offs among different strategies, helping to guide the choice of the most suitable system based on the specific application, performance, and practicality.

### 3.4. Host–Guest Encapsulation

**Figure 9:**
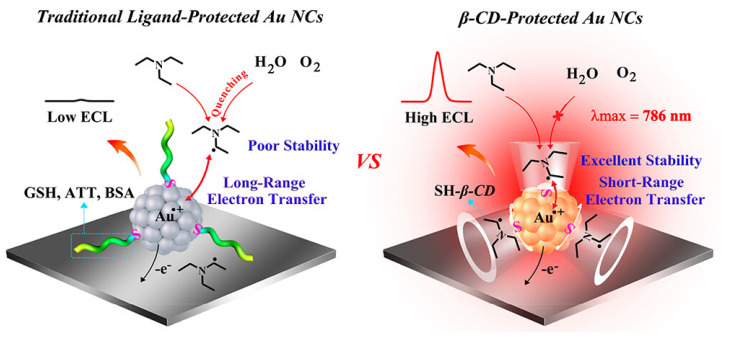
Schematic illustration comparing traditional ligand-protected Au NCs such as ATT-Au NCs, BSA-Au NCs, and GSH-Au NCs without a shielding effect (**left**) and *β-CD-protected* Au NCs with a ligand-based shielding effect (**right**). Copyright 2023 American Chemical Society [66].

The stability of co-reactant radical intermediates is crucial for achieving efficient Au NCs-based ECL systems. However, in aqueous environments, these intermediates often exhibit poor stability, which significantly hampers ECL efficiency. Additionally, unprotected active co-reactant intermediates can be quenched by factors such as dissolved oxygen and water, further contributing to reduced ECL performance [114,115]. Addressing this issue is thus important for improving the reliability and effectiveness of Au NCs-based ECL systems. Recently, Yuan et al. reported the ligand-based shielding effect of *β*-cyclodextrin-protected Au NCs (*β*-CD-Au NCs) on the ECL efficiency of the clusters in the presence of TEA as a co-reactant [66]. As shown in Figure 9, the shielding effect of the *β*-CD ligand enables the stabilization of TEA**^•^** intermediates within the *β*-CD cavity, preventing their exposure to the environment so they are not quenched by dissolved oxygen, water, or other factors. The *β*-CD ligand features a hydrophobic cavity that can host TEA**^•^** intermediates through host-guest chemistry. Additionally, the shielding effect of the *β*-CD ligand shortens the electron transfer pathway by encapsulating TEA**^•^** intermediates within the cavity of *β*-CD-Au NCs even without extensive chemical modification of the clusters. These effects led to a higher ECL efficiency of *β*-CD-Au NCs compared to traditional ligand-protected Au NCs; 321 times greater than BSA-Au NCs, 153 times greater than ATT-Au NCs, and 19 times greater than GSH-Au NCs. The *β*-CD-Au NCs were thus utilized as emitters in a “signal off” ECL sensing platform to detect noradrenaline as a model target, achieving a low detection limit of 0.91 nM. This work is important because it emphasizes the key role of ligands in improving the stability of active co-reactant intermediates for high-efficiency Au NCs-based ECL systems.

## 4. Conclusions

Since co-reactants are a main component of practical co-reactant ECL pathways in Au NCs-based ECL systems and thus play a crucial role in the ECL efficiency of Au NCs, many innovative co-reactant engineering strategies have been developed to enhance the ECL performance of these systems. So far, we have reviewed some recent studies among many innovative ones, including the design of new co-reactant molecules, co-reactant accelerators, structural integration, and host-guest strategies to address the challenges of Au NCs-based ECL systems. Despite numerous promising developments, several challenges remain in the field of ECL systems utilizing Au NCs. Future research could be directed to enhancing the environmental compatibility of co-reactants, streamlining synthetic procedures, and reducing hazardous substances without compromising ECL performance. Additionally, the integration of advanced functional materials, such as nanozymes and bio-nanomaterials, holds potential for further enhancing the sensitivity and selectivity of the Au NCs-based ECL systems. Furthermore, incorporating computational modeling and data-driven artificial intelligence (AI) tools may facilitate the rational design and optimization of co-reaction parameters. By providing comprehensive insights into the Au NCs-based ECL systems and further developing co-reactant engineering strategies for Au NCs, we believe that, despite existing challenges, this field will continue to inspire significant advancements in the future. This progression is expected to advance from laboratory-scale studies to large-scale and practical applications of the Au NCs-based ECL systems.

## Figures and Tables

**Table 1:** Co-reactants in different Au NCs-based ECL systems.

Co-Reactants	Luminophores	Φ_ECL_ (%)	Ref.
Triethylamine (TEA)	GSH-Au NCs	0.42	[68]
	BSA-Au NCs	9.8	[68]
	Ox-Met-Au NCs	66.1	[68]
	ATT-Au NCs	78	[69]
	Co^2+^-Au NCs	33.8	[70]
	Hydrogel-confined Au NCs	95	[52]
	Discrete Au NCs	0.41	[52]
Tripropylamine (TPrA)	Au_25_ NCs	103	[71]
	Au_12_-Ag_13_ NCs	400 times higher (vs. Ru(bpy)_3_^2+^/TPrA)	[72]
	Arg-ATT-Au NCs	67.02	[73]
Triethanolamine (TEOA)	NAC/Cys-Au NCs	N/A	[63]
	Met-Au NCs	75 times higher (vs. BSA-Au NCs)	[62]
Potassium persulfate	Met-Au NCs	2.33	[74]
	BSA-Au NCs	0.33	[74]
	NAC-Au NCs	4.11	[75]
	Zn^2+^-MHA-Au NCs	10.54	[76]
Benzoyl peroxide (BPO)	Au NCs	32	[59]
Hydrazine	BSA-Au NCs	N/A	[77]
*N*,*N*-diethylethylenediamine (DEDA)	LA-Au NCs	17 times higher (vs. Ru(bpy)_3_^2+^/TPrA)	[78]
Tris(3-aminoethyl)amine (TAEA)	Pd@CuO-Au NCs	N/A	[79]
	Polypeptide-biomineralize Au NCs	N/A	[80]
*N*,*N*-disopropylethylenediamine (DPEA)	Au-DPEA NCs	2.1 times higher (vs. Au NCs)	[81]
*N*,*N*-diisopropylethylamine (DIPEA)	*β*-CD-Au NCs	728	[82]
EDTA	LA-Au NCs	N/A (Higher at pH 7.4 than at more basic and acidic pHs)	[83]
HEPES	LA-Au NCs	N/A (Optimal at physiological pH)	[84]

Note: All ECL efficiency values (Φ_ECL_) and ECL intensity comparisons (e.g., ‘17 times higher’) are taken directly from the original studies according to the calculations and methods described therein. The part after ‘vs’ indicates the system used for comparison in the original work. ‘N/A’ means the original studies did not provide a numerical ECL efficiency.

## Data Availability

No new data were created or analyzed in this study. Data sharing is not applicable to this article.
